# SIRTUIN 1 ISOFORMS DIFFERENTIALLY IMPACT MITOCHONDRIAL GENE EXPRESSION AND FUNCTION IN MUSCLE CELLS

**DOI:** 10.1093/geroni/igad104.2495

**Published:** 2023-12-21

**Authors:** Xiaomin Zhang, Pankaj Patyal, Ambika Verma, Shakshi Sharma, Gohar Azhar, Fathima Ameer, Yingni Che, Jeanne Wei

**Affiliations:** University of Arkansas for Medical Sciences, Little Rock, Arkansas, United States; University of Arkansas for Medical Sciences, Little Rock, Arkansas, United States; University of Arkansas for Medical Sciences, Little Rock, Arkansas, United States; University of Arkansas for Medical Sciences, Little Rock, Arkansas, United States; University of Arkansas for Medical Sciences, Little Rock, Arkansas, United States; University of Arkansas for Medical Sciences, Little Rock, Arkansas, United States; University of Arkansas for Medical Sciences, Little Rock, Arkansas, United States; University of Arkansas for Medical Sciences, Little Rock, Arkansas, United States

## Abstract

**Background:**

Alternative splicing (AS) is a mechanism that generates multiple mRNA transcripts and protein isoforms from a single gene, thereby increasing transcriptomic and proteomic diversity. The spliced isoforms commonly lack one or more exons, which may have similar or even opposite functions. Alternatively spliced isoforms tend to increase during aging. The sirtuin-1 gene contains multiple exons and undergoes alternative splicing, which generates multiple isoforms. In this study, we assessed the impact of three sirtuin-1 isoforms on mitochondrial gene expression and function in muscle cells.

**Methods:**

Three sirtuin-1 isoforms (V1, V2, V3) were subcloned into expression vectors. Muscle cell lines (H9C2 and C2C12) were transfected with sirtuin-1 isoforms, respectively. Microscopic images were obtained using a Nikon microscope. Gene expression was determined by quantitative RT-PCR and Western blotting. The mitochondrial function was determined with a Seahorse XFe96 Analyzer. Results and conclusions The sirtuin-1 V1 isoform significantly increased the oxygen consumption rate (OCR) and decreased glycolysis (ECAR) in muscle cells, while V2 and V3 isoforms had slight or no significant effect on OCR and ECAR. V1 isoform was localized in the nucleus, whereas V2 and V3 were localized in the cytoplasm. Sirtuin-1 isoforms differentially impacted mitochondrial complex genes, including NDUFS1, NDUFV1, NDUFV2, NDUFA5. Our data indicate that the domain loss changed sirtuin-1 isoform subcellular localization, differentially impacted mitochondrial gene expression, and affected mitochondrial function. The age-related change in the expression of sirtuin-1 isoforms could affect cardiac and skeletal muscle function in aging and senescence. Further exploration of the sirtuin-1 isoforms’ functions is warranted.

